# Risk factors for major cardiovascular adverse events and the effects of sacubitril/valsartan in patients with acute myocardial infarction after Percutaneous Coronary Intervention

**DOI:** 10.12669/pjms.41.6.10819

**Published:** 2025-06

**Authors:** Yang Peng, Yuxiang Wen, Han Wei

**Affiliations:** 1 Yang Peng, Department of Cardiology, The First Affiliated Hospital of Yangtze University, Jingzhou 434000, Hubei, China; 2 Yuxiang Wen, Department of Cardiology, The First Affiliated Hospital of Yangtze University, Jingzhou 434000, Hubei, China; 3 Han Wei, Department of Cardiology, The First Affiliated Hospital of Yangtze University, Jingzhou 434000, Hubei, China

**Keywords:** Acute myocardial infarction, Emergency percutaneous coronary intervention, Major cardiovascular adverse events, Sacubitril/Valsartan

## Abstract

**Objective:**

To investigate the risk factors for major cardiovascular adverse events (MACEs) in patients with acute myocardial infarction (AMI) after percutaneous coronary intervention (PCI), and observe the effect of sacubitril/valsartan (SV) on these events.

**Methods:**

This was a retrospective study. One hundred and twenty-five patients with AMI undergoing PCI admitted to The First Affiliated Hospital of Yangtze University from January 2022 to August 2024 were included and divided into MACEs group(n=54) and non-MACEs group(n=71) according to whether MACEs occurred one week after PCI. Univariate/multivariate logistics regression analysis of risk factors for MACEs. Patients were divided into SV group(n=65) and non-SV group(n=60) according to whether taking SV, adverse events, blood lipids, hemorheology, and cardiac function were compared using Chi-square or t-test.

**Results:**

The incidence of MACEs was 43.20% (54/125). Logistics regression analysis showed that an age of ≥70 years, smoking history, hyperlipidemia, anemia, hypertension grade≥2, and time from onset to balloon dilation ≥7 hours were independent risk factors for MACEs. The incidence of MACEs was 30.78% in SV group, lower than non-SV group (56.67%) (χ^2^=8.528, P=0.003). After 6 months of treatment, the levels of TG, TC, and LDL-C were significantly decreased, and the level of HDL-C was significantly increased in SV group compared with non-SV group (P<0.05). The improvement in whole blood high/low shear viscosity, plasma viscosity, the level of fibrinogen,LVEDD, LVEBD, and LVEF was better in SV group than non-SV group(P<0.05).

**Conclusion:**

Use of SV can improve blood lipids, hemorheology, and cardiac function, and reduce the incidence of post-PCI MACEs in these patients.

## INTRODUCTION

Acute myocardial infarction (AMI) is a common cardiac emergency encountered in clinical practice and mainly caused by acute ischemia and hypoxia of the cardiovascular system, with severe stenosis of coronary arteries as the main clinical manifestations. The mortality rate of AMI is up to 5% in China.^1^ Although the clinical outcomes of AMI have been significantly improved with the advancement of drug treatment and direct percutaneous coronary intervention (PCI), it remains an important disease threatening human health and life.^2^ The main treatment options for AMI currently available include thrombolytic therapy, PCI, and coronary artery bypass grafting (CABG). PCI is effective to quickly clear narrow or even occluded coronary artery lumens, rebuild blood flow, and improve myocardial blood perfusion, making it the most rapidly developing and highly recommended treatment method in recent years.^3^ With the rapid development of PCI, the mortality rate of AMI has significantly decreased.

However, some patients may experience major cardiovascular adverse events (MACEs), including recurrent MI, AP, arrhythmia, and HF, due to poor perfusion or reperfusion injury in the short term after PCI,^4^ which affects the clinical efficacy of PCI and greatly impairs the quality of life and long-term prognosis of these patients. Therefore, prevention of post-PCI MACEs is of great importance. However, the influencing factors of post-PCI MACEs are complex. How to detect and intervene the risk factors for MACEs in advance and how to reduce the incidence of post-PCI MACEs in AMI patients have become the key to the improved long-term prognosis of AMI. SV is the first approved angiotensin receptor neprilysin inhibitor and has been widely used in clinical practice. However, the effect of SV on post-PCI MACEs in patients with AMI has been rarely reported. Therefore, this study was designed to explore the risk factors for post-PCI MACEs and the effect of SV on these events in patients with AMI undergoing PCI.

## METHODS

This was a retrospectively study. A total of 125 eligible patients with AMI who were hospitalized in The First Affiliated Hospital of Yangtze University between January 2022 to August 2024 and treated with PCI were retrospectively selected, and divided into the MACE group (n=54) and the non-MACE group (n=71) according to the incidence of MACEs occurred within one week after operation.

### Ethical Approval:

The study was approved by the Institutional Ethics Committee of the First Affiliated Hospital of Yangtze University (No.:KY2024-058-01; Date: August 23, 2024), and written informed consent was obtained from all participants.

### Inclusion criteria:

- Who met the diagnostic criteria for AMI and were definitely diagnosed by coronary angiography.
- Who had the indications for emergency PCI treatment and underwent emergency PCI for the first time, with a successful surgery.
- With an age of 18-75 years old, and completed PCI within 24 hours.
- Who signed the informed consent form with complete clinical data.

### Exclusion criteria:

- With severe liver and kidney dysfunction, infectious diseases, and mental disorders.
- Complicated with malignant tumors.
- Whose condition improved after thrombolytic therapy and did not receive PCI.
- Who were not able to cooperate with treatment with incomplete clinical data.

### PCI and medical treatment:

- Pre-operative treatment: Patients were given oral aspirin 300 mg and ticagrelor 180mg;
- Surgical procedure: Unfractionated heparin were intravenously injected for anticoagulation. Transradial or femoral artery puncture, and a guiding catheter was inserted into the narrowed coronary artery through the vascular access. A balloon was inserted into the narrowed area under the guidance of the guidewire to dilate the blood vessel. The dilation pressure and time were adjusted as appropriate, and the balloon was withdrawn after the coronary artery stenosis was relieve, after withdrawing the balloon, implant a drug-eluting stent; and
- Postoperative treatment, including lipid-lowering, blood pressure lowering, and anticoagulation drugs, were given. SV was orally administered at a dose of 100 mg/time, twice a day.

### Methods and Outcome Measures:

- MACEs, including recurrent MI, arrhythmia, HF, and SCD, in the patients within one week after PCI was collected;
- General and clinical data of two groups of patients were collected, including physical examination, laboratory tests, echocardiogram, and PCI treatment. For patients complicated with hypertension, hypertension was graded using the following criteria: Grade 1: systolic blood pressure of 140-159 mmHg, and diastolic blood pressure of 90-99 mmHg; Grade 2: systolic blood pressure of 160-179 mmHg, and diastolic blood pressure of 100-109 mmHg; and Grade 3: systolic blood pressure ≥180 mmHg, and diastolic blood pressure ≥110mmHg;
- General and clinical data were compared between the two groups using univariate analysis, and factors with statistically significant differences were included in a multivariate logistic regression model to analyze the risk factors for MACEs;
- Patients were divided into the SV group and the non-SV group according to the use of SV, and the differences in MACEs, hemorheology, and cardiac function parameters between the two groups were compared at admission and after six months of treatment.

### Statistical Analysis:

Data were analyzed using SPSS 22.0 software. Measurement data were presented as mean ± standard deviation (*x̄* ± *s*) and t-test was used; and enumeration data were presented as n (%), and χ² test was used. Related risk factors were analyzed using Logistic regression analysis, and survival was analyzed using the Cox proportional hazards regression analysis. Differences with a p<0.05 were considered statistically significant.

## RESULTS

A total of 125 patients were included in this study, including 74 males and 51 females, with a mean age of 68.34±9.78 years (range: 42-82 years), and a time from onset to balloon dilatation of 6.76±1.01 hours (4.5-9.2 hours). The incidence of post-PCI MACEs was 43.20% (54/125), with four patients with SCD, 15 with HF, 15 with AP, and 20 with dyspnea.

The age, smoking history, incidence of complications, preoperative blood pressure, LVEF, number of patients with no-reflow after PCI, and time from onset to balloon dilatation were increased in the MACE group compared with those in the non-MACE group, and the differences were statistically significant (P<0.05) (Table-I). Multiple logistic regression analysis was conducted using the incidence of MACEs in one week after PCI as the dependent variable and factors which were statistically significant in univariate analysis as independent variables. The results showed that an age of ≥70 years, smoking history, hyperlipidemia, anemia, hypertension ≥grade 2, and time from onset to balloon dilation ≥7 hours were risk factors for post-PCI MACEs in patients with AMI (Table-II).

**Table-I:** Univariate analysis of influencing factors of post-PCI MACEs.

Factors	The MACE group (n=54)	The non-MACE group (n=71)	t/χ²	P
Age(years)	71.70±10.26	65.79±8.62	3.499	0.001
Sex(M /F)	31/23	43/28	0.126	0.722
Smoking history (Yes/No)	40/14	33/38	9.614	0.002
Drinking history (Yes/No)	30/24	40/31	0.008	0.930
Complications				
Old MI	22	8	14.607	<0.001
Hyperlipidemia	21	12	7.632	0.006
Anemia	32	12	24.126	<0.001
Coronary heart disease	28	11	18.889	<0.001
Diabetes	31	14	18.910	<0.001
Hypertension Grade 1	6	20	5.418	0.020
Hypertension Grade 2	12	6	4.719	0.030
Hypertension Grade 3	16	4	13.141	<0.001
LVEF >40	11	39	15.264	<0.001
PCI				
Number of implanted stents	2.30±0.74	2.23±0.66	0.564	0.574
Stent diameter	2.29±0.25	2.19±0.46	1.353	0.179
No-reflow after PCI	24	16	6.766	0.009
Time from onset to PCI	7.52±0.84	6.17±0.68	9.908	<0.001

**Table-II:** Logistics analysis of the influencing factors of post-PCI MACEs.

Factors	β	Standard error	Wald value	P	OR(95%CI)
Age ≥70 years	4.148	1.121	13.706	0.000	0.002-0.142
Smoking history	1.208	0.570	4.481	0.034	0.098-0.914
Hyperlipidemia	5.454	1.684	10.492	0.001	8.618-6335.751
Anemia	2.423	1.049	5.339	0.021	0.011-0.692
Hypertension Grade ≥2	2.149	1.041	4.264	0.039	0.015-0.897
Time from onset to balloon dilation ≥7hrs	2.483	1.231	4.072	0.044	1.074-133.618

In the 125 patients enrolled, 65 patients took SV, and 60 did not. MACEs occurred in 20 patients in the SV group, with an incidence of 30.78%, including one patient with SCD, and 34 patients in the non-SV group, with an incidence rate of 56.67%, including one patient with SCD. The difference between the two groups was statistically significant (*χ*^2^=8.528, P=0.003). There were no statistically significant differences in the levels of TC, TG, LDL-C, and HDL-C between the two groups before treatment (P>0.05); and after six months of treatment, the levels of TG, TC, and LDL-C in both groups decreased, and the levels of these parameters were reduced in the SV group compared with those in the non-SV group, while the level of HDL-C increased in the two groups, and the level was increased in the SV group compared with that in the non-SV group. The differences between the two groups were statistically significant (P<0.05) (Table-III).

**Table-III:** Comparison of blood lipid parameters between the two groups before and after treatment(x̄ ± S).

Groups	n	TG(mmol/L)		TC(mmol/L)		LDL-C(mmol/L)		HDL-C(mmol/L)	
		Before treatment	After 6 months of treatment	Before treatment	After 6 months of treatment	Before treatment	After 6 months of treatment	Before treatment	After 6 months of treatment
The SV group	64	5.29±0.66	2.86±0.69	8.10±0.89	5.64±0.85	4.70±0.57	2.59±0.63	1.29±0.40	2.48±0.51
The non-SV group	57	5.38±0.75	3.60±0.66	7.87±0.75	6.14±0.69	4.68±0.69	2.91±0.66	1.28±0.41	1.91±0.49
*t*		0.675	6.028	1.461	3.537	0.143	2.749	0.162	6.204
*P*		0.501	<0.001	0.147	0.001	0.887	0.007	0.871	<0.001

No significant differences in whole blood high/low shear viscosity, plasma viscosity and fibrinogen were observed between the two groups before treatment (P>0.05); and after 6 months of treatment, the whole blood high/low shear viscosity, plasma viscosity and fibrinogen decreased in the two groups, and the improvement of these parameters was better in the SV group than that in the non-SV group, with statistically significant differences(P<0.05) (Table-IV).

**Table-IV:** Comparison of hemorheological parameters between the two groups before and after treatment(x̄ ± S).

Groups	n	Whole blood high shear viscosity(mpa/s)		Whole blood low shear viscosity(mpa/s)		Plasma viscosity(mpa/s)			Fibrinogen(g/L)
		Before treatment	After 6 months of treatment	Before treatment	After 6 months of treatment	Before treatment	After 6 months of treatment	Before treatment	After 6 months of treatment
The SV group	64	9.46±0.66	4.21±0.55	14.41±2.39	8.20±2.07	2.29±0.25	1.42±0.74	5.29±0.56	2.34±0.74
The non-SV group	57	9.39±0.21	4.83±0.21	14.79±2.50	10.15±3.13	2.30±0.25	1.80±0.37	5.17±0.27	3.47±0.65
*t*		0.696	8.005	0.841	4.080	0.188	3.530	1.491	8.912
*P*		0.487	<0.001	0.402	<0.001	0.851	<0.001	0.139	<0.001

There were no significant differences in LVEDD, LVEBD and LVEF between the two groups before treatment (P>0.05); and after treatment, the levels of LVEDD, LVEBD and LVEF in the two groups were improved compared with those before treatment (P<0.05), and the improvement of LVEDD, LVEBD and LVEF in the SV group was significantly better than that in the non-SV group, with statistically significant differences(P<0.05) (Table-V).

**Table-V:** Comparison of cardiac function parameters between the two groups before and after treatment(x̄ ± S).

Groups	n	LVEF(%)		LVEDD(mm)		LVEBD(mm)	
		Before treatment	After treatment	Before treatment	After treatment	Before treatment	After treatment
The SV group	64	41.36±4.20	57.34±4.74	60.23±3.67	48.19±4.58	56.06±3.63	43.84±4.19
The non-SV group	57	40.56±3.94	52.37±3.61	59.39±3.62	54.19±3.57	55.42±3.42	48.28±3.49
*t*		1.075	6.436	1.278	7.967	0.997	6.282
*P*		0.285	<0.001	0.204	<0.001	0.321	<0.001

## DISCUSSION

The results of this study showed that age ≥ 70 years, smoking history, concomitant hyperlipidemia, hypertension grade ≥ 2, anemia, and time from onset to balloon dilation ≥ 7 hours were all risk factors for postoperative cardiovascular adverse events in patients. Among these risk factors, age is an uncontrollable factor. Relevant literature shows that the incidence of AMI is gradually increasing in recent years,^5^ and its onset characteristics have a trend towards younger age. However, the functions of various organs in elderly people are severely degraded compared to young and middle-aged people, and they are more prone to various cardiovascular, pulmonary, liver and kidney diseases. Patients with AMI complicated with multiple diseases have poor tolerance and are more likely to develop MACEs after undergoing PCI.^6^

Smoking can affect the respiratory system, leading to abnormal lung function, and also have adverse effects on the safety of PCI surgery for AMI patients.^7^ Hyperlipidemia can accumulate and form plaques on the inner wall of blood vessels, reduce vascular elasticity, damage the myocardium, increase myocardial oxygen consumption, and weaken anticoagulant effects; With the increase of blood pressure, coronary atherosclerosis can damage the vascular endothelial function and form coronary thrombus, and then induce a variety of MACEs.^8^ Anemia leads to insufficient blood supply to the myocardium, increased myocardial oxygen consumption, increased respiratory rate, and increased susceptibility to arrhythmia^.9^ If the blocked blood vessel can be reopened in a timely manner after myocardial infarction, it can restore blood supply to the affected myocardium to the greatest extent possible and restore normal function. If the ischemia time is prolonged, the possibility of recovery of the affected myocardium decreases, leading to a higher risk of MACEs in patients after PCI.^10^

PCI is currently an important method for treating AMI,^11^ but there is a high risk of multiple MACEs occurring after PCI. In clinical practice, a series of measures can be taken to prevent the occurrence of MACEs, but currently there is still a lack of methods for preventing and treating MACEs after PCI.^12^ The results of this study showed that the incidence of MACEs after PCI in the medication group was 30.78%, which was lower than the 56.67% in the non-medication group (χ 2=8.528, P=0.003). Indicating that taking sacubitril valsartan can reduce the incidence of MACEs in AMI patients after PCI.

The mechanism is that sacubitril valsartan can maintain circulating levels of natriuretic peptide, block the renin angiotensin aldosterone system and sympathetic nervous system to exert anti heart failure, improve heart function and cardiac remodeling effects.^13^ TC and LDL-C can damage the intima of blood vessels, form plaques, and cause luminal stenosis. AMI patients often have dyslipidemia.^14^ The results of this study showed that after 6 months of treatment, the levels of TG, TC, and LDL-C in both groups decreased, and the taking group was lower than the non-taking group (P<0.05), indicating that sacubitril and valsartan can effectively improve lipid metabolism. This is because sacubitril and valsartan can promote the normal generation and differentiation of adipocytes, improve lipid metabolism status, and achieve normal lipid metabolism processes.^15^

AMI patients often have abnormal hemorheological indicators such as increased blood viscosity and slow blood flow. Improving hemorheological indicators is an important measure for preventing and treating AMI.^16^ The results of this study showed that after six months of treatment, the whole blood high/low shear viscosity, plasma viscosity, and fibrinogen of patients significantly decreased, and the improvement effect was better in the treatment group (P<0.05), indicating that sacubitril valsartan can improve the hemorheological indicators of AMI patients. This is because sacubitril valsartan regulates water sodium balance and dilates blood vessels by acting on the renin angiotensin aldosterone system, thereby regulating changes in hemorheological indicators.^17^

The cardiac function of patients with AMI is often lower than normal, and improving cardiac function is the key to preventing and treating AMI.^18^ The results of this study showed that after treatment, the levels of LVEDD, LVEBD, and LVEF in both groups of patients improved compared to before treatment, and the improvement in the medication group was more significant (P<0.05). The analysis suggests that sacubitril and valsartan can reduce myocardial cell apoptosis, prevent ventricular fibrosis, and alleviate myocardial vascular remodeling.^19^

## CONCLUSION

The incidence of post-PCI MACEs in patients with AMI is associated with an age of ≥70 years, smoking, hyperlipidemia, anemia, hypertension, and time from onset to balloon dilation ≥7 hours. Treatment with SV can improve the parameters of blood lipids, hemorheology, and cardiac function and reduce the incidence of post-PCI MACEs in patients with AMI. However, this was a single center retrospective study with a relatively small sample size, which limited the conclusions of the study, and future large-scale studies with long-term follow-up are needed to further explore the risk factors for post-PCI MACEs in patients with AMI and the effect of SV on these MACEs.

### Authors’ Contributions:

**HW** and **YP:** Carried out the studies, data collection, drafted the manuscript, are responsible and accountable for the accuracy or integrity of the work.

**YW:** Analyzed, performed the statistical analysis and participated in its design.

All authors have read and approved the final manuscript.
